# Supplementary material for: How many categories are there in crossmodal correspondences? A study based on exploratory factor analysis
Source: PLoS One. 2023 Nov 14;18(11):e0294141. doi: 10.1371/journal.pone.0294141 (PMC10645324; doi:10.1371/journal.pone.0294141)

Supporting Information

S1 Fig. Examples of the visual stimuli used in Experiment 1 and 2.

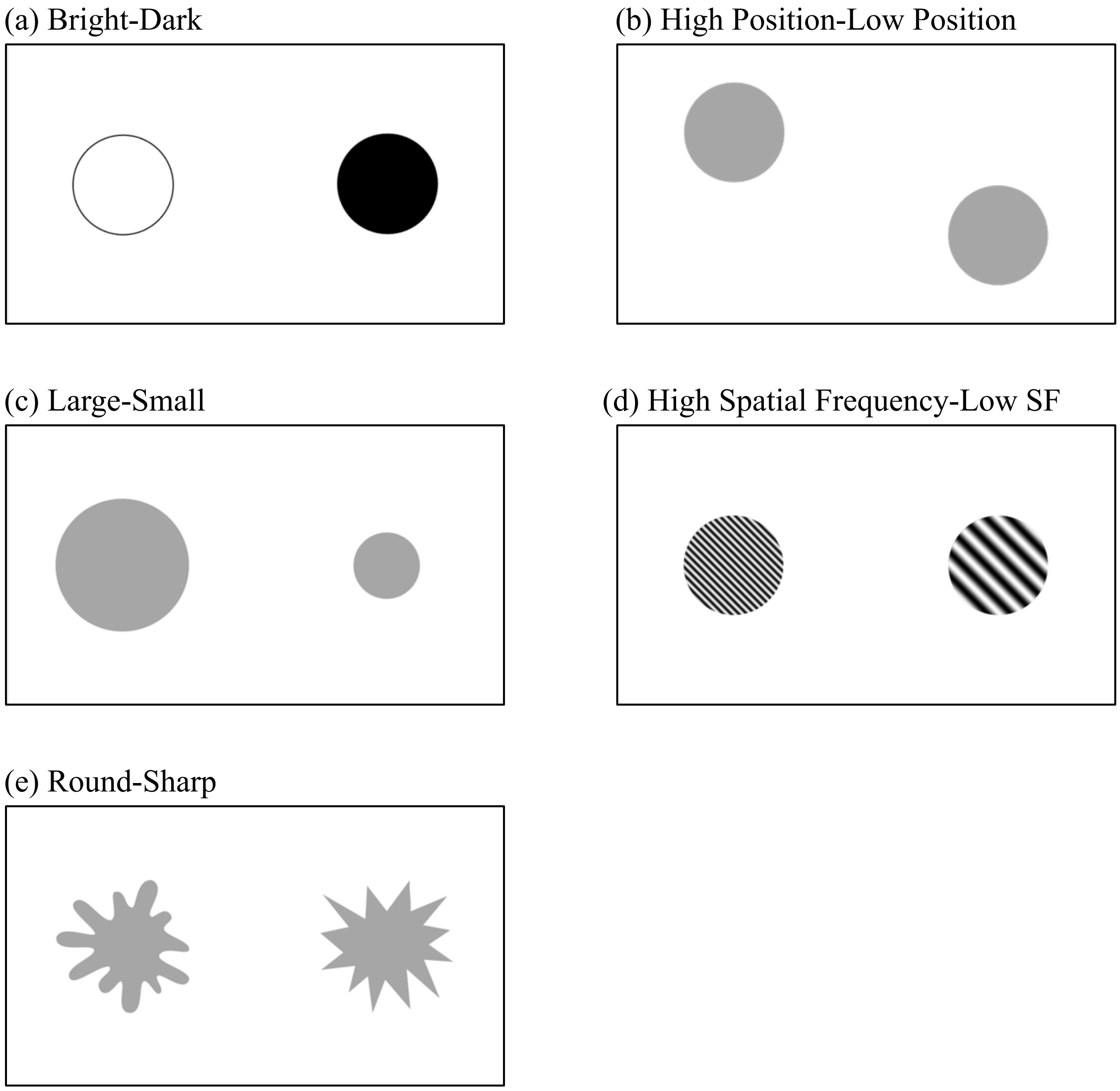

Supplement: S1 Fig — (PDF) [file pone.0294141.s001.pdf]
