# Supplementary material for: How many categories are there in crossmodal correspondences? A study based on exploratory factor analysis
Source: PLoS One. 2023 Nov 14;18(11):e0294141. doi: 10.1371/journal.pone.0294141 (PMC10645324; doi:10.1371/journal.pone.0294141)
Supplement: Attachment — Submitted filename: Response to Reviewers.docx [file pone.0294141.s003.docx]

Response to Reviewer #1
We greatly appreciate your invaluable support for our manuscript. Here, we summarize how we addressed your suggestions and revised the manuscript.

**Comment #1**

**Mudd (1963) didn’t investigate CCs as such. Rather they investigated spatial qualities associated with tones, that subsequent researchers have interpreted in terms of CCs.**

Response:

As you pointed out, Mudd (1963) investigated spatial features associated with pure tones in four dimensions. To accurately introduce the study, we have revised the description (L51 ~ 56).

**Comment #2:**

**Smith is not an author of Marks et al.’s (1987) paper, but write a separate commentary on their article.**

Response:

Thank you for pointing this out. We have corrected the citation of the relevant text and References (L60 ~ 61, ref 8).

**Comment #3:**

**There are still problems with references. Newly-Added ref 1, you need to make clear these are (Eds.) of handbook not authors of it.**

Response:

Thank you for your attention to detail. We have corrected the mistake in the Reference according to the PLOS reference style (ref 1).

**Comment #4:**

**Sadaghiani, S., Maier, J. X., & Noppeney, U. (2009). Natural, metaphoric, and linguistic auditory direction signals have distinct influences on visual motion processing. Journal of Neuroscience, 29, 6490-6499. May also be relevant in showing different neural substrates for linguistic and non-linguistic correspondences**

Response:

Thank you for sharing the important paper. We have cited this paper in the relevant text in the General Discussion (L515).

**Comment #5:**

**p. 21 “For example, the correspondence between pitch and elevation could be explained either by the internalization of natural statistics or the use of the same words” I am not sure it is necessarily either/or, various correspondences might also have some contribution from both?**

Response:

We meant to say that pitch and elevation could be explained at least in two ways. As you pointed out, we did not mean that the two explanations/underlying processes were exclusive. To make this clear, we have modified the description (L103).
